# Computing Offloading Based on TD3 Algorithm in Cache-Assisted Vehicular NOMA–MEC Networks

**DOI:** 10.3390/s23229064

**Published:** 2023-11-09

**Authors:** Tianqing Zhou, Ming Xu, Dong Qin, Xuefang Nie, Xuan Li, Chunguo Li

**Affiliations:** 1 School of Information Engineering, East China Jiaotong University, Nanchang 330013, Chinalixuan@ecjtu.edu.cn (X.L.); 2 School of Information Engineering, Nanchang University, Nanchang 330031, China; 3 School of Information Science and Engineering, Southeast University, Nanjing 210096, China; chunguoli@seu.edu.cn

**Keywords:** TD3, MEC, NOMA, vehicular networks, edge cache, computation offloading, resource allocation

## Abstract

In this paper, in order to reduce the energy consumption and time of data transmission, the non-orthogonal multiple access (NOMA) and mobile edge caching technologies are jointly considered in mobile edge computing (MEC) networks. As for the cache-assisted vehicular NOMA–MEC networks, a problem of minimizing the energy consumed by vehicles (mobile devices, MDs) is formulated under time and resource constraints, which jointly optimize the computing resource allocation, subchannel selection, device association, offloading and caching decisions. To solve the formulated problem, we develop an effective joint computation offloading and task-caching algorithm based on the twin-delayed deep deterministic policy gradient (TD3) algorithm. Such a TD3-based offloading (TD3O) algorithm includes a designed action transformation (AT) algorithm used for transforming continuous action space into a discrete one. In addition, to solve the formulated problem in a non-iterative manner, an effective heuristic algorithm (HA) is also designed. As for the designed algorithms, we provide some detailed analyses of computation complexity and convergence, and give some meaningful insights through simulation. Simulation results show that the TD3O algorithm could achieve lower local energy consumption than several benchmark algorithms, and HA could achieve lower consumption than the completely offloading algorithm and local execution algorithm.

## 1. Introduction

With the rapid development of information and communication technologies, the data traffic generated by vehicles (mobile devices, MDs) has also significantly increased [1]. For wireless communication networks, more spectrum resources are required for data traffic transmission [2,3,4,5]. In addition, higher computing power is required by MDs for supporting large amounts of task calculation. However, due to the limited battery capacity of MDs, it may be challenging to process these computation tasks for them. By deploying edge computing servers at base stations (BSs), mobile edge computing (MEC) can support MDs in processing tasks at the adjacent edge servers [6,7]. Compared with cloud computing (CC), which requires tasks to be uploaded to a remote cloud, MEC can provide additional computing resources for MDs within its coverage area and thus reduce their computing overhead [8,9,10,11,12,13,14].

Although the edge servers can reduce the computing overhead of MDs by providing more computing resources, the extra time and energy consumption caused by offloading tasks through wireless channels cannot be ignored, especially for high-size computation tasks. In order to further reduce the time and energy consumption caused by offloading tasks, edge caching technology is also introduced into MEC networks. By caching tasks of MDs at edge servers in advance, the overhead caused by offloading tasks could be greatly reduced [15,16,17,18,19].

To upload tasks from MDs to edge servers, orthogonal multiple access (OMA) is often used, but it may be greatly challenging to provide a high transmission rate and support massive connections. As another type of resource utilization management, non-orthogonal multiple access (NOMA) technologies can let multiple users share the same frequency bands, achieve higher spectral efficiency and support extensive connections [20,21,22,23]. It is evident that NOMA is a good type of resource utilization management for reducing the cost of task transmission in MEC networks.

Although the application of caching and NOMA technologies in MEC networks can reduce time and energy consumption, such a framework will make the design of computation offloading and edge caching schemes more complex. To the best of our knowledge, until now, how to jointly perform the device association, computation offloading, edge caching, subchannel selection and resource allocation is still an important and open topic in cache-assisted NOMA–MEC networks.

### 1.1. Related Work

So far, a lot of work has been conducted on joint computation offloading and resource optimization in NOMA–MEC networks. In [20], joint radio and computation resource allocation was optimized to maximize the offloading energy efficiency in NOMA–MEC-enabled IoT networks, and a solution based on a multi-layer iterative algorithm was proposed. In [21], local computation resource, offloading ratio, uplink transmission time and power and subcarrier assignment were jointly optimized to minimize the sum of weighted energy consumed by users in NOMA–MEC networks, and some effective iterative algorithms were designed for single-user and multi-user cases. In [24], joint task offloading, power allocation and computing resource allocation were optimized to achieve delay minimization using a deep reinforcement learning (DRL) algorithm in NOMA–MEC networks. In [25], the joint optimization of offloading decisions, local and edge computing resource allocation and power and subchannel allocation were realized to minimize energy consumption in heterogeneous NOMA–MEC networks, and an effective iterative algorithm was designed. In [26], power and computation resource allocations were jointly optimized to minimize overall computation and transmission delay for massive MIMO and NOMA-assisted MEC systems, and a solution based on an interior-point algorithm was given. In [27], the channel resource allocation and computation offloading policy were jointly optimized to minimize the sum of weighted energy and latency in NOMA–MEC networks, and some efficient solutions were found using a DRL algorithm based on actor–critic and deep Q-network (DQN) methods.

To further reduce the offloading time and energy consumption, edge caching technology is introduced into conventional MEC networks. Such a framework has attracted more and more attention. In [28], the offloading and caching decisions, uplink power and edge computing resources were jointly optimized to minimize the sum of weighted local processing time and energy consumption in two-tier cache-assisted MEC networks, and a distributed collaborative iterative algorithm was proposed. In [29], a problem of adaptive request scheduling and cooperative service caching was studied in cache-assisted MEC networks. After formulating the optimization problems as partially observable Markov decision process (MDP) problems, an online DRL algorithm was proposed to improve the service hitting ratio and latency reduction rate. In [30], optimal offloading and caching strategies were established to minimize overall delay and energy consumption of all regions using a deep deterministic policy gradient (DDPG) framework in cache-assisted multi-region MEC networks. In [31], joint MD association and resource allocation were performed to minimize the sum of MDs’ weighted delay in heterogeneous cellular networks with MEC and edge caching functions, and an effective iterative algorithm was developed using coalitional game and convex optimization theorems. In [32], to minimize the content transmission delay in vehicular edge computing networks, a cooperative vehicular edge computing and caching scheme based on asynchronous federated and deep reinforcement learning was proposed to predict the popular content and the optimal cooperative caching location of the content. In [33], to reduce the cost of the cloud service center through the asynchronous advantage actor–critic algorithm, the offloading decision, service caching and resource allocation strategies were jointly optimized in the three-tier mobile cloud–edge computing structure combining computation offloading and service caching mechanisms. In [34], a logistic function-based service reliability probability (SRP) estimation model was built, and the average SRP maximization problem of a virtual machine-based edge computing server was studied for such a model. At last, a low-complexity heuristic alternative optimization algorithm was proposed.

To enhance spectral efficiency and support massive connections, NOMA technology has attracted increasing attention in cache-assisted MEC networks. In [35], the multi-agent deep deterministic policy gradient method was used to dynamically optimize the user association, power control and cache placement of BSs and satellites to improve the network energy efficiency in a NOMA-enabled satellite integrated with a terrestrial network scenario. In [36], joint optimization of offloading and caching decisions and computation resource allocation was performed to maximize long-term reward in cache-assisted NOMA–MEC networks under the predicted task popularity, and single-agent and multi-agent Q-learning algorithms were proposed to find feasible solutions. In [37], joint optimization of offloading and caching decisions was performed to minimize the system delay in cache-assisted NOMA–MEC networks, and a multi-agent DQN algorithm was used for finding efficient solutions under the predicted popularity. In [38], local task processing time was minimized by jointly optimizing offloading and caching decisions and the allocation of edge computing resources and uplink power in cache-assisted NOMA–MEC networks with single BS, and the blocking successive upper-bound minimization method was utilized to achieve efficient solutions.

Although the framework of cache-assisted (vehicular) NOMA–MEC networks can greatly reduce the task processing time and energy consumption and support massive connections, there exist very few relevant efforts. Unlike the above-mentioned work, we jointly optimize the edge computing resource allocation, subchannel selection, device association, offloading and caching decisions for the cache-assisted vehicular NOMA–MEC networks with multiple BSs, minimizing the energy consumed by MDs under time and resource constraints. In addition, unlike existing efforts, we develop an effective dynamic joint computation offloading and task-caching algorithm based on the twin-delayed deep deterministic policy gradient algorithm (TD3) to find efficient solutions, named the TD3-based offloading (TD3O) algorithm.

### 1.2. Contribution and Organization

In this paper, we jointly optimize the edge computing resource allocation, subchannel selection, device association, offloading and caching decisions in cache-assisted vehicular NOMA–MEC networks, minimizing the energy consumed by MDs under time and resource constraints. Specifically, the main contributions and work of this paper can be listed as follows.

- Edge computing resource allocation, subchannel selection, device association, computation offloading and edge caching are jointly performed in cache-assisted vehicular NOMA–MEC networks. To the best of our knowledge, work that concerns subchannel selection is a new investigation for cache-assisted vehicular NOMA–MEC networks with multi-server scenarios. Meanwhile, as far as this problem is concerned, the goal is to minimize the energy consumed by MDs under the constraints of time, computing resources, caching capacity, the number of MDs associated with each BS and the number of MDs associated with each subchannel. As far as we know, such an optimization problem is a new concentration in cache-assisted vehicular NOMA–MEC networks.
- We design effective algorithms to find feasible solutions to the formulated problem. Considering that the formulated problem is in a mixed-integer, nonlinear, multi-constraint form, a simple map between actions and actual policies in a conventional twin-delayed deep deterministic policy gradient (TD3) algorithm cannot be well applied. In addition, too large an action space will cause the TD3 algorithm to fail to search for correct actions and thus fail to converge. In view of these concerns, we develop an effective TD3O algorithm integrating with the AT algorithm to solve the formulated problem. Moreover, in order to solve this problem in a non-iterative manner, an effective heuristic algorithm (HA) is also designed.
- Performance analyses of the designed algorithms. Some analyses are made for the computation complexity and convergence of the designed algorithms in detail. In addition, some meaningful simulation analyses are also made by introducing other benchmark algorithms for comparison, and some good results and insights are achieved.

The rest of the paper is organized as follows. Section 2 introduces the system model. Section 3 formulates a problem of minimizing local energy consumption in cache-assisted vehicular NOMA–MEC networks. Section 4 designs the HA and TD3O algorithm. Section 5 gives the computation complexity and convergence analyses for the designed algorithms. Section 6 investigates the performance of the designed algorithms through simulation. Section 7 gives conclusions and discussions.

## 2. System Model

### 2.1. Network Model

**Figure 1:**
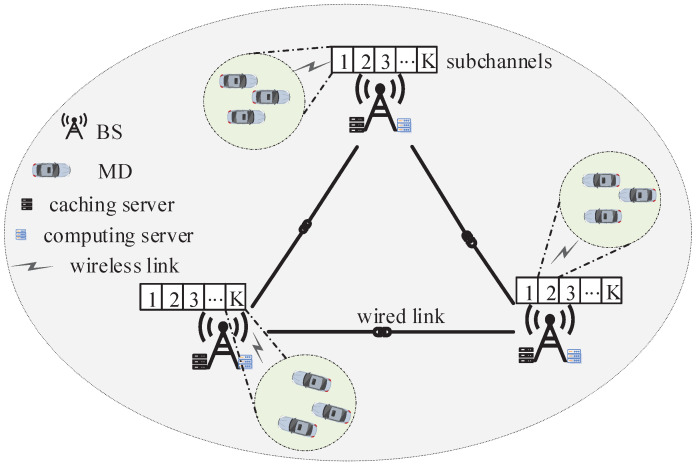
Cache-assisted vehicular NOMA–MEC networks.

Figure 1 shows the cache-assisted vehicular NOMA–MEC networks. In such network, there exist *M* MDs, and the index set of them is denoted as M={1,2,⋯,M}; *B* BSs are deployed, and the index set of them is given by $I=\left\{1,2,\cdots ,I\right\}$. In addition, each BS is equipped with one edge computing server and one edge caching server, and these BSs connect to each other through wired links. We assume that each MD has one computation task at any timeslot, which can be processed by itself, its associated BS or another auxiliary BS selected by this associated BS. When tasks have been cached at the BSs used for processing them, they do not need to be uploaded to these BSs; when the associated BSs have not cached tasks, MDs need to upload tasks to these BSs; when the auxiliary BSs have not cached tasks, the associated BSs need to upload tasks to their selected auxiliary BSs.

Assume that the association index between MD *m* and BS *i* is $x_{m,i}\in \left\{0,1\right\}$, where X = $\left\{x_{m,i}\left|\forall m\in M,\forall i\in I\right.\right\}$. $x_{m,i}=1$ if MD *m* is associated with BS *i*, otherwise $x_{m,i}=0$. In addition, we assume that the caching index of the task of MD *m* at BS *i* is denoted as $y_{m,i}\in \left\{0,1\right\}$, where $Y=\left\{y_{m,i}|\forall m\in M,\forall i\in I\right\}$. $y_{m,i}=1$ if the task of MD *m* is cached at BS *i*, otherwise $y_{m,i}=0$. We also assume that the offloading (execution) index of the task of MD *m* at BS *i* is denoted as $u_{m,i}$, where $U=\left\{u_{m,i}|\forall m\in M,\forall i\in I\right\}$. $u_{m,i}=1$ if the task of MD *m* is executed at BS *i*, otherwise $u_{m,i}=0$. At last, we assume that the association index between MD *m* and subchannel *k* of BS *i* is denoted as $z_{m,i,k}$, where $Z=\left\{z_{m,i,k}|\forall m\in M,\forall i\in I,\forall k\in K\right\}$. If $x_{m,i}\left(1-y_{m,i}\right)\left(1-y_{m,\bar{i}}\right)u_{m,\bar{i}}=1$ or $x_{m,i}\left(1-y_{m,i}\right)u_{m,i}$ = 1 under $\bar{i}\neq i$, MD *m* can select (be associated with) some subchannel *k* of BS *i*, which means $z_{m,i,k}=1$. Otherwise, the subchannel *k* of BS *i* cannot be selected by MD *m*, which means $z_{m,i,k}=0$.

### 2.2. Communication Model

In this paper, the system bandwidth *W* is divided into *K* subchannels with equal bandwidth, which are indexed by $K=\left\{1,2,\cdots ,K\right\}$. These subchannels can be shared by different MDs through NOMA. Significantly, each MD can occupy at most one subchannel, the number of MDs selecting each subchannel cannot exceed the upper limit ρ, and the number of MDs associated with any BS that need to upload tasks should be less than or equal to the number of subchannels *K* [37].

As revealed in [39], the successive interference cancellation (SIC) technology in NOMA technology can effectively reduce the interference between MDs in the same subchannel. The channel gains of MDs sharing the same subchannel of a BS should be sorted in descending order at first, and then the uplink NOMA signals received by this BS can be decoded in this order. We assume that $M_{k}^{SC}$ is the set of MDs selecting subchannel *k*, and $o_{m,i,k}$ represents the sequence number of channel gain between MD *m* and BS *i* on subchannel *k*. When MD *i* and MD *m* access the subchannel *k* of BS *i* simultaneously and the channel gain $h_{j,i,k}$ between MD *j* and BS *i* on subchannel *k* is lower than the channel gain $h_{m,i,k}$ between MD *m* and BS *i* on subchannel *k*, $o_{j,i,k}<o_{m,i,k}$ is satisfied. Then, the signal of MD *m* is decoded but the signal of MD *j* will be treated as noise. Therefore, when MD *m* selects subchannel *k* of BS *i*, its uplink data rate $r_{m,i,k}$ can be given by

(1) $$r_{m,i,k}=W\log_{2}\left(1+p_{m}h_{m,i,k}/\left(\Gamma_{m,i,k}+\sigma^{2}\right)\right)/K,$$

where $\Gamma_{m,i,k}=\sum_{j\in M_{k}^{sc}/\left\{m\right\}:o_{j,i,k}<o_{m,i,k}}p_{j}h_{j,i,k}$ is the interference caused by other MDs (excluding MD *m*) sharing subchannel *k* of BS *i* through NOMA; $p_{m}$ is the transmission power of MD *m*; $\sigma^{2}$ is the noise power. When MD *m* is decoded, it is no longer regarded as interference in the subchannel, and the device with the maximum channel gain among the remaining MDs in the current subchannel is decoded in the same way until all MDs of the current subchannel are decoded.

### 2.3. Caching and Offloading Models

In this paper, we assume that any MD *m* has a time-sensitive task denoted as $L_{m}=\left\{d_{m},c_{m},\tau_{m}^{\max}\right\}$ at each timeslot, where $d_{m}$ is the data size of the task of MD *m*, $c_{m}$ is the number of CPU cycles required to complete a one-bit task, and $\tau_{m}^{\max}$ is the maximum task processing time of MD *m*.

**Figure 2:**
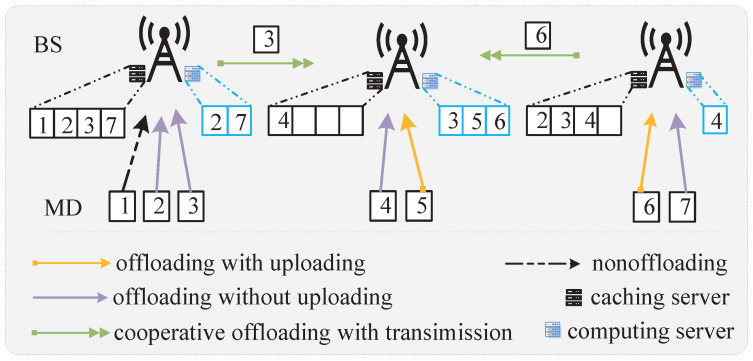
Caching and offloading models.

Figure 2 illustrates the caching and offloading models. At each timeslot, BSs precache the tasks for processing at the next timeslot. When MD *m* is associated with BS *i*, it first checks whether the associated BS has cached the corresponding task. If $\sum_{i\in I}u_{m,i}=0$, the task of MD *m* is calculated by itself, e.g., MD 1 in Figure 2. If $x_{m,i}y_{m,i}u_{m,i}=1$, the task of MD *m* can be directly calculated at its associated BS *i*, and the results will be fed back from BS *i* to MD *m*, e.g., MD 2 in Figure 2. If $x_{m,i}y_{m,i}\left(1-y_{m,\bar{i}}\right)u_{m,\bar{i}}=1$, the task of MD *m* is offloaded from its associated BS *i* to another auxiliary BS $\bar{i}\neq i$ for computing through a wired link, e.g., MD 3 in Figure 2. If $x_{m,i}y_{m,i}y_{m,\bar{i}}u_{m,\bar{i}}=1$, the task of MD *m* can be directly calculated at auxiliary BS $\bar{i}\neq i$, e.g., MD 4 in Figure 2. If $x_{m,i}\left(1-y_{m,i}\right)u_{m,i}=1$, the task of MD *m* will be offloaded to its associated BS *i* for computing, e.g., MD 5 in Figure 2. If $x_{m,i}\left(1-y_{m,i}\right)\left(1-y_{m,\bar{i}}\right)u_{m,\bar{i}}=1$, the task of MD *m* first needs to be offloaded to its associated BS *i*, and then it is transmitted from this BS to another auxiliary BS $\bar{i}\neq i$ for computing through a wired link, e.g., MD 6 in Figure 2. If $x_{m,i}\left(1-y_{m,i}\right)y_{m,\bar{i}}u_{m,\bar{i}}=1$, the task of MD *m* can be directly calculated at an auxiliary BS $\bar{i}\neq i$, e.g., MD 4 in Figure 2.

#### 2.3.1. Local Computing

If $\sum_{i\in I}u_{m,i}=0$ is satisfied, the task of MD *m* should be executed locally, and the processing time and energy consumption are, respectively, given by

(2) τmloc=cmdmcmdmfmlocfmloc,

(3) $$\varepsilon_{m}^{\mathrm{loc}}=\xi c_{m}d_{m}{\left(f_{m}^{\mathrm{loc}}\right)}^{2},$$

where $f_{m}^{\mathrm{loc}}$ is the computing capacity of MD *m*, and ξ is an energy-consumption coefficient depending on the hardware architecture.

#### 2.3.2. Task Transmission

If $x_{m,i}\left(1-y_{m,i}\right)\left(1-y_{m,\bar{i}}\right)u_{m,\bar{i}}=1$ or $x_{m,i}\left(1-y_{m,i}\right)u_{m,i}=1$ are satisfied under $\bar{i}\neq i$, the task of MD *m* should be, respectively, uploaded to BS $\bar{i}$ or *i* for execution through NOMA. Then, the uploading time and energy consumption of MD *m*, respectively, are given by

(4) $$\tau_{m}^{\mathrm{trs}}=\sum_{i\in I}\sum_{k\in K}z_{m,i,k}d_{m}/r_{m,i,k},$$

(5) $$\varepsilon_{m}^{\mathrm{trs}}=p_{m}\tau_{m}^{\mathrm{trs}}.$$

In addition, if $x_{m,i}\left(1-y_{m,i}\right)\left(1-y_{m,\bar{i}}\right)u_{m,\bar{i}}=1$ or $x_{m,i}y_{m,i}\left(1-y_{m,\bar{i}}\right)u_{m,\bar{i}}=1$ is satisfied under $\bar{i}\neq i$, the task of MD *m* should be transmitted from its associated BS *i* to an auxiliary BS $\bar{i}$ through a wired link, and the corresponding time is given by

(6) τmbh=dmdmrbhrbh,

where $r^{\mathrm{bh}}$ is the backhualing rate between any two BSs.

In this paper, we mainly concentrate on the energy consumption of MDs but not the energy consumed by BSs. In addition, the downlink transferring time of results is often ignored since they are fairly small [40].

#### 2.3.3. Edge Computing

When MD *m* executes its task at BS *i*, the task processing time at this BS can be given by

(7) τm,iexe=cmdmcmdmfm,ifm,i,

where $f_{m,i}$ is the computing capacity allocated to MD *m* by BS *i*.

#### 2.3.4. Task Processing Time and Energy Consumption

Then, the total time used for processing the task of MD *m* can be given by

(8) $$\begin{array}{cc} \tau_{m}^{\mathrm{tot}}= & \sum_{i\in I}(\left(1-\sum_{i\in I}u_{m,i}\right)\tau_{m}^{\mathrm{loc}}\\ \; & +x_{m,i}\left(1-y_{m,i}\right)\sum_{\bar{i}\in I\setminus \left\{i\right\}}u_{m,\bar{i}}\left(1-y_{m,\bar{i}}\right)\tau_{m}^{\mathrm{trs}}\\ \; & +x_{m,i}\left(1-y_{m,i}\right)\sum_{\bar{i}\in I\setminus \left\{i\right\}}u_{m,\bar{i}}\left(1-y_{m,\bar{i}}\right)\tau_{m}^{\mathrm{bh}}\\ \; & +x_{m,i}\left(1-y_{m,i}\right)\sum_{\bar{i}\in I\setminus \left\{i\right\}}u_{m,\bar{i}}\tau_{m,\bar{i}}^{\mathrm{exe}}\\ \; & +x_{m,i}y_{m,i}\sum_{\bar{i}\in I\setminus \left\{i\right\}}u_{m,\bar{i}}\left(1-y_{m,\bar{i}}\right)\tau_{m}^{\mathrm{bh}}\\ \; & +x_{m,i}y_{m,i}\sum_{\bar{i}\in I\setminus \left\{i\right\}}u_{m,\bar{i}}\tau_{m,\bar{i}}^{\mathrm{exe}}\\ \; & +x_{m,i}\left(1-y_{m,i}\right)u_{m,i}\left(\tau_{m}^{\mathrm{trs}}+\tau_{m,i}^{\mathrm{exe}}\right)\\ \; & +x_{m,i}y_{m,i}u_{m,i}\tau_{m,i}^{\mathrm{exe}}). \end{array}$$

On the right side of the equality sign in (8), the first item represents the local executing time; the second item is the time used for uploading the task from MD *m* to the associated BS *i*, which does not cache this task and further transmits it to auxiliary BS for computing; the third item is the time used for transmitting the task from the associated BS *i* to another auxiliary BS, where these two BSs do not cache this task; the fourth item is the time used for executing the task of MD *m* at an auxiliary BS, where the associated BS does not cache this task; the fifth item is the time used for transmitting the task from the associated BS *i* to another auxiliary BS, where the associated BS caches this task but the auxiliary BS does not; the sixth item is the time used for executing the task of MD *m* at an auxiliary BS, where the associated BS caches this task; the seventh item includes the time used for transmitting the task from MD *m* to the associated BS *i*, which does not cache this task, and the time used for executing the task of MD *m* at this BS; the eighth item is the time used for executing the task of MD *m* at the associated BS *i*, which caches this task.

Then, the total local energy consumption used for processing the task of MD *m* can be given by

(9) $$\begin{array}{cc} \varepsilon_{m}^{\mathrm{tot}}= & \sum_{i\in I}(\left(1-\sum_{i\in I}u_{m,i}\right)\varepsilon_{m}^{\mathrm{loc}}\\ \; & +x_{m,i}\left(1-y_{m,i}\right)\sum_{\bar{i}\in I\setminus \left\{i\right\}}u_{m,\bar{i}}\left(1-y_{m,\bar{i}}\right)\varepsilon_{m}^{\mathrm{trs}}\\ \; & +x_{m,i}\left(1-y_{m,i}\right)u_{m,i}\varepsilon_{m}^{\mathrm{trs}}), \end{array}$$

on the right side of equality sign in (9), the first item represents the local executing energy consumption; the second item is the energy consumption caused by offloading the task from MD *m* to its associated BS *i*, which further transmits this task to auxiliary BS $\bar{i}\neq i$ for computing; the third item is the energy consumption caused by transmitting the task from MD *m* to the associated BS *i*, which does not cache this task.

## 3. Problem Formulation

Until now, we have formulated a problem of minimizing local energy consumption at each given period. Specifically, under the constraints of time, computing resources, caching capacity, the number of MDs associated with each BS and the number of MDs associated with each subchannel, we jointly optimized the edge computing resource allocation, subchannel selection, device association, offloading and caching decisions to minimize the energy consumed by MDs in cache-assisted vehicular NOMA–MEC networks. Mathematically, this is formulated as

(10) $$\begin{array}{cc} P1: & \min_{X,Y,U,Z,F}\sum_{m\in M}\varepsilon_{m}^{\mathrm{tot}}\\ s.t.\; & C_{1}:\tau_{m}^{\mathrm{tot}}\leq \tau_{m},\forall m\in M,\\ \; & C_{2}:\sum_{i\in I}x_{m,i}=1,\forall m\in M,\\ \; & C_{3}:\sum_{i\in I}\sum_{k\in K}z_{m,i,k}\leq 1,\forall m\in M,\\ \; & C_{4}:\sum_{m\in M}\sum_{k\in K}z_{m,i,k}\leq K,\forall i\in I,\\ \; & C_{5}:\sum_{i\in I}u_{m,i}\leq 1,\forall m\in M,\\ \; & C_{6}:x_{m,i}\in \left\{0,1\right\},\forall m\in M,\forall i\in I,\\ \; & C_{7}:y_{m,i}\in \left\{0,1\right\},\forall m\in M,\forall i\in I,\\ \; & C_{8}:z_{m,i,k}\in \left\{0,1\right\},\forall m\in M,\forall i\in I,\forall k\in K,\\ \; & C_{9}:u_{m,i}\in \left\{0,1\right\},\forall m\in M,\forall i\in I,\\ \; & C_{10}:\sum_{m\in M}y_{m,i}d_{m}\leq \vartheta_{i},\forall i\in I,\\ \; & C_{11}:\sum_{m\in M}\sum_{i\in I}z_{m,i,k}\leq \rho ,\forall k\in K,\\ \; & C_{12}:\sum_{m\in M}u_{m,i}f_{m,i}\leq f_{i}^{\mathrm{BS}},\forall i\in I, \end{array}$$

where $F=\left\{f_{m,i}\left|\forall m\in M,\forall i\in I\right.\right\}$; the constraint $C_{1}$ gives the maximum task processing time of MD *m*; $C_{2}$ and $C_{6}$ indicate that any MD *m* can select only one BS; $C_{3}$ and $C_{8}$ indicate that any MD *m* can occupy at most one subchannel; $C_{4}$ and $C_{8}$ mean that the number of MDs selecting any BS that needs to upload tasks should be less than or equal to the number of subchannels; $C_{5}$ and $C_{9}$ mean that any MD *m* can select at most one BS to execute its task; $C_{7}$ and $C_{10}$ indicate that the data size of tasks cached at BS *i* does not exceed the caching capacity $\vartheta_{i}$ of this BS; $C_{8}$ and $C_{11}$ show that the number of MDs selecting a subchannel cannot exceed its upper limit; $C_{7}$ and $C_{12}$ reveal that the total computing capacity allocated to MDs by BS *i* cannot exceed the computing capacity of this BS.

## 4. Algorithm Design

As previously mentioned, the optimization problem P1 refers to minimizing local energy consumption within a given period. In view of this, we adopt the DRL algorithm to solve it. DRL is based on MDP, which implements the environment-based output of agent policy in MDP through neural networks, maximizing certain rewards. Considering that the overestimation of some conventional DRL algorithms (e.g., DQN and DDPG), the TD3 algorithm has been widely advocated because it can overcome well the problems of the above algorithms and achieve more stable output [41,42]. The main features of the TD3 algorithm are adding a new neural network and reducing the training frequency of the network based on DDPG.

The problem P1 has both continuous and discrete variables, and the solution space formed by the combination of all variables is very large, which is not a suitable scenario for the DQN algorithm to solve discrete space problems. Therefore, we use the TD3 algorithm, which can solve the continuous solution space problems. Considering that a simple mapping between only the decision of the algorithm and the actual strategy will fail to achieve convergence because of there being too many feasible strategies and the inability to search for the correct one, we develop an effective TD3O algorithm integrated with the AT algorithm to solve the problem P1.

### 4.1. MDP Used for Describing Problem P1

Considering that the optimization problem P1 needs to be tackled within a given period, in order to apply TD3O to the problem P1, such a period is divided into *T* timeslots and denoted as T={1,2,⋯,T}. Furthermore, the problem of joint computing offloading, task caching and resource allocation is described as a MDP, the state space, action space and reward function are defined as follows.

**❶** State space: At each timeslot, the state space contains the information used for decisions made by the network. Here, the state $s_{t}$ at timeslot *t* can be denoted as $s_{t}=\left\{\bar{D}\left(t+1\right),\bar{Y}\left(t\right)\right\}$. The detailed definitions can be found as follows.

- $\bar{D}\left(t+1\right)=\left\{{\bar{d}}_{m}\left(t+1\right)\left|\forall m\in M\right.\right\}$ are the standardized data sizes of tasks of MDs at timeslot t+1, where
  (11) $${\bar{d}}_{m}\left(t\right)=\frac{d_{m}\left(t\right)-d^{\min}\left(t\right)}{d^{\max}\left(t\right)-d^{\min}\left(t\right)},$$
  $d^{\min}\left(t\right)$ is the minimum data size of the tasks of all MDs at timeslot *t*, and $d^{\max}$ is the maximum data size of the tasks of all MDs at timeslot *t*.
- $\bar{Y}\left(t\right)=\left\{{\bar{y}}_{m}\left(t\right)\left|\forall m\in M\right.\right\}$ are the task caching decision factors at BSs at timeslot *t*, where ${\bar{y}}_{m}\in \left[0,1\right]$.

**❷** Action space: At each timeslot, the action space refers to the decisions made by the network according to the state $s_{t}$. The action $a_{t}$ at timeslot *t* can be denoted as $a_{t}=\left\{\bar{X}\left(t\right),\bar{Y}\left(t+1\right),\bar{Z}\left(t\right),\bar{U}\left(t\right),\bar{F}\left(t\right)\right\}$. Specifically:

- $\bar{X}\left(t\right)=\left\{{\bar{x}}_{m}\left(t\right)\left|\forall m\in M\right.\right\}$ are the association decision factors of MDs at timeslot *t*, where ${\bar{x}}_{m}\in \left[0,1\right]$.
- $\bar{Y}\left(t+1\right)=\left\{{\bar{y}}_{m}\left(t+1\right)\left|\forall m\in M\right.\right\}$ are the caching decision factors at timeslot *t* for the next timeslot.
- $\bar{Z}\left(t\right)=\left\{{\bar{z}}_{i}\left(t\right)\left|\forall i\in I\right.\right\}$ are the subchannel allocation decision factors of BSs at timeslot *t*, where ${\bar{z}}_{i}\in \left[0,1\right]$.
- $\bar{U}\left(t\right)=\left\{{\bar{u}}_{m}\left(t\right)\left|\forall m\in M\right.\right\}$ are the offloading decision factors of MDs at timeslot *t*, where ${\bar{u}}_{m}\in \left[0,1\right]$.
- $\bar{F}\left(t\right)=\left\{{\bar{f}}_{m}\left(t\right)\left|\forall m\in M\right.\right\}$ are the computing resource allocation factors of MDs at timeslot *t*, where ${\bar{f}}_{m}\in \left[0,1\right]$.

It is noteworthy that the dimensions of the above-mentioned state and action spaces have been greatly reduced compared to the actual ones. The actual state and action spaces can be achieved by executing an AT algorithm in the following parts.

**❸** Reward: Considering that the goal of problem P1 is to minimize local energy consumption and the constraints $C_{1}$ and $C_{10}$ cannot be strictly satisfied in the DRL-based iteration procedure, the reward $w_{t}$ at timeslot *t* is given by

(12) $$w_{t}=-\omega_{1}\sum_{m\in M}\varepsilon_{m}^{\mathrm{tot}}\left(t\right)-\omega_{2}\phi \left(t\right)-\omega_{3}\varphi \left(t\right),$$

where $\phi \left(t\right)=\sum_{m\in M}\max(\tau_{m}^{\mathrm{tot}}\left(t\right)-\tau_{m},0)$ is the penalty function added for guaranteeing the constraint $C_{1}$; $\varphi \left(t\right)=\sum_{i\in I}\max(\sum_{m\in M}y_{m,i}\left(t\right)d_{m}\left(t\right)-\vartheta_{i}\left(t\right),0)$ is the penalty function introduced for guaranteeing the constraint $C_{10}$; $\omega_{1}$ is the energy-consumption discount factor; $\omega_{2}$ and $\omega_{3}$ are penalty coefficients.

When the network obtains action $a_{t}$ according to the state $s_{t}$, the state space will obtain the next state $s_{t+1}$ according to the action $a_{t}$. Specifically, the task-caching decisions of BSs can be directly achieved from $Y\left(t+1\right)$ in $a_{t}$. Therefore, the total return of minimizing long-term local energy consumption within *T* timeslots can be given by

(13) $$R=\sum_{t\in T}\gamma w_{t},$$

where γ is the reward discount factor satisfying $\gamma \in \left(0,1\right)$.

### 4.2. TD3O Algorithm

The TD3 algorithm is an actor-critic-based framework that comprises the policy (μ) network, critic (Q) network and their corresponding target networks and updates the network parameters using gradient algorithms. It is characterized by using two critic networks and two critic target networks in the design of critic networks. The TD3 algorithm is often divided into two parts consisting of experience collection and training. In the phase of collecting experience, a new action $a_{t}$ can be generated by adding random Gaussian noise into the output of the policy network at the state $s_{t}$, i.e.,

(14) $$a_{t}=\mu \left(s_{t},\theta^{\mu }\right)+{\bar{\sigma }}^{2}.$$

where $\theta^{\mu }$ is the parameter of the policy network and ${\bar{\sigma }}^{2}$ is the additive Gaussian noise.

After that, the environment is rewarded with $w_{t}$ and the next state $s_{t+1}$ can be achieved according to the state and action $\left(s_{t},a_{t}\right)$. To enable the algorithm to obtain better decisions through past experience-assisted training, we put the quadruple $\left(s_{t},a_{t},w_{t},s_{t+1}\right)$ into the experience replay buffer as a historical experience. In the training process, a certain number of quadruples are randomly selected from the experience replay buffer for training. Since the TD3 algorithm consists of policy and critic networks, the training part of the network is relatively independent, so it is divided into the following two parts.

#### 4.2.1. Training Policy Network

**Figure 3:**
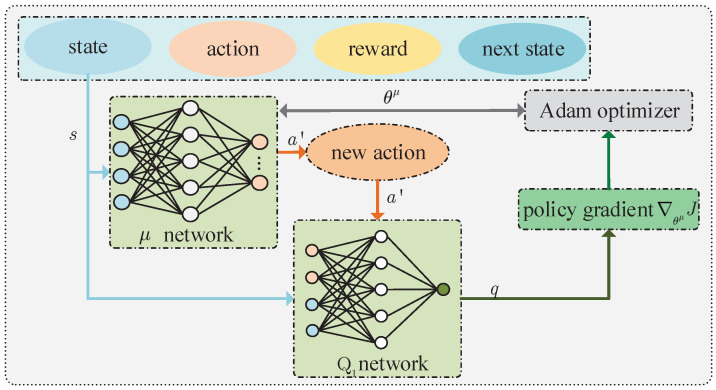
Training policy network.

The training process of the policy network is shown in Figure 3. In the training phase, *N* quadruples are extracted from the experience replay buffer and denoted as $N=\left\{1,2,\cdots ,N\right\}$. For any quadruple n∈N, the policy network outputs a new action ${a^{\prime }}_{n}=\mu \left(s_{n},\theta^{\mu }\right)$ according to the state $s_{n}$. It should be noted that the policy ${a^{\prime }}_{n}$ is different from $a_{n}$ existing in the experience replay buffer. After $s_{n}$ and ${a^{\prime }}_{n}$ are inputted into any critic network (e.g., critic $Q_{1}$ network), such network outputs $q_{n}=Q_{1}\left(s_{n},\mu \left(s_{n},\theta^{\mu }\right),\theta^{Q_{1}}\right)$, where $\theta^{Q_{1}}$ is the parameter of the critic $Q_{1}$ network. After achieving all $q_{n}$, their mathematical expectation is given by

(15) $$J\left(\theta^{\mu }\right)=E\left[Q_{1}\left(S,\mu \left(S,\theta^{\mu }\right),\theta^{Q_{1}}\right)\right],$$

where $S=\left\{s_{n}|n\in N\right\}$. Then, the policy gradient of function *J* with respect to $\theta^{\mu }$ can be given by

(16) $$\nabla_{\theta^{\mu }}J=E\left[\nabla_{A}Q_{1}\left(S,A,\theta^{Q_{1}}\right)\nabla_{\theta^{\mu }}\mu \left(S,\theta^{\mu }\right)\right],$$

where $A=\left\{a_{n}|n\in N\right\}$.

Significantly, the calculated gradient requires gradient clipping, which can avoid skipping the optimal solution because the gradient is too large. The calculated policy gradients will be used to update the parameters of the policy networks. We assume that the learning rate of the policy network is $\beta^{\mu }$, and use the adaptive moment (Adam) estimation commonly used in DRL to obtain the optimal $\theta^{\mu }$ [43].

#### 4.2.2. Training Critic Network

**Figure 4:**
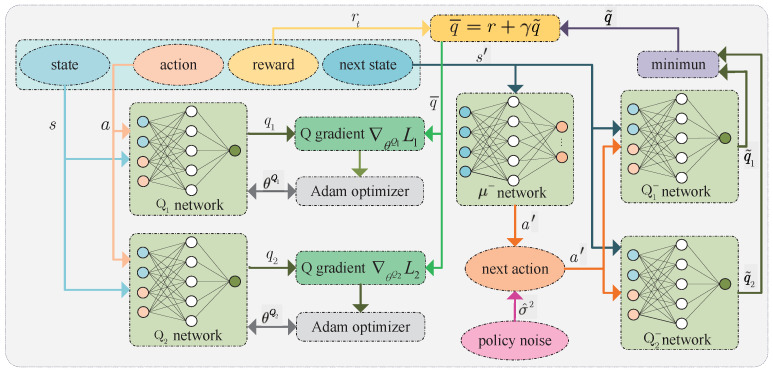
Training critic network.

Figure 4 shows the training process of the critic network. During the critic network training, the policy at the next time is first estimated through the state at the next time by the policy target($\mu^{-}$) network, i.e., ${a^{\prime }}_{n}=\mu^{-}\left({s^{\prime }}_{n},\theta^{\mu^{-}}\right)+{\hat{\sigma }}^{2}$, where ${\hat{\sigma }}^{2}$ is policy noise, that is, trimmed additive Gaussian noise. Then, the action ${a^{\prime }}_{n}$ and the state ${s^{\prime }}_{n}$ are used as the input of the critic target ($Q_{1}^{-})$ network and critic target ($Q_{2}^{-}$) network, where $\theta^{Q_{1}^{-}}$ and $\theta^{Q_{2}^{-}}$ are their parameters. After that, these two networks output ${\tilde{q}}_{n,1}$ and ${\tilde{q}}_{n,2}$, respectively. Next, the approximation of Q value is ${\bar{q}}_{n}=r_{n}+\gamma {\tilde{q}}_{n}$ achieved using Behrman equation, where ${\tilde{q}}_{n}=\min\left({\tilde{q}}_{n,1},{\tilde{q}}_{n,2}\right)$. At the same time, the action $a_{n}$ and the state $s_{n}$ are used as the input of the critic $Q_{1}$ network and critic $Q_{2}$ network, where $\theta^{Q_{1}}$ and $\theta^{Q_{2}}$ are their parameters. After that, these two networks output $q_{n,1}$ and $q_{n,2}$. At last, for all ${\bar{q}}_{n}$, according to the theorem of mean squared error (MSE), the expectation function of the squared loss between $Q_{1}\left(S,A,\theta^{Q_{1}}\right)$ and $\bar{Q}$ is

(17) $$L_{1}\left(\theta^{Q_{1}}\right)=0.5E\left[{\left(Q_{1}\left(S,A,\theta^{Q_{1}}\right)-\bar{Q}\right)}^{2}\right],$$

and the expectation function of the squared loss between $Q_{2}\left(S,A,\theta^{Q_{2}}\right)$ and $\bar{Q}$ is given by

(18) $$L_{2}\left(\theta^{Q_{2}}\right)=0.5E\left[{\left(Q_{2}\left(S,A,\theta^{Q_{2}}\right)-\bar{Q}\right)}^{2}\right],$$

where $\bar{Q}=\left\{{\bar{q}}_{n}|n\in N\right\}$. Then, the gradient of the loss function $L_{1}\left(\theta^{Q_{1}}\right)$ with respect to the parameter $\theta^{Q_{1}}$ is

(19) $$\nabla_{\theta^{Q_{1}}}L_{1}=E\left[\left(Q_{1}\left(S,A,\theta^{Q_{1}}\right)-\bar{Q}\right)\nabla_{\theta^{Q_{1}}}Q_{1}\left(S,A,\theta^{Q_{1}}\right)\right],$$

and the gradient of the loss function $L_{2}\left(\theta^{Q_{2}}\right)$ with respect to the parameter $\theta^{Q_{2}}$ is given by

(20) $$\nabla_{\theta^{Q_{2}}}L_{2}=E\left[\left(Q_{2}\left(S,A,\theta^{Q_{2}}\right)-\bar{Q}\right)\nabla_{\theta^{Q_{2}}}Q_{2}\left(S,A,\theta^{Q_{2}}\right)\right].\;$$

Similar to calculating the policy gradient, the gradient clipping needs to be performed after calculating the gradients using (19) and (20). In addition, $\beta^{Q}$ is the learning rate of the critic network, and the parameters of the two critic networks are updated using the Adam algorithm. Certainly, the parameters of critic target networks also need to be updated using the soft update method, i.e.,

(21) $$\theta^{\mu^{-}}=\lambda \theta^{\mu }+\left(1-\lambda \right)\theta^{\mu^{-}},$$

(22) $$\theta^{Q_{1}^{-}}=\lambda \theta^{Q_{1}}+\left(1-\lambda \right)\theta^{Q_{1}^{-}},$$

(23) $$\theta^{Q_{2}^{-}}=\lambda \theta^{Q_{2}}+\left(1-\lambda \right)\theta^{Q_{2}^{-}},$$

where λ is the learning rate of target networks.

It is noteworthy that a lower network updating frequency is adopted in this paper. We assume that the update interval of the critic network is $t^{\mathrm{cti}}$ and the update interval between the policy and critic networks is $t^{\mathrm{pti}}$. The critic networks are trained many times to ensure the stability of Q value. After that, the policy network can be updated. The detailed procedure of the TD3O algorithm is summarized in Algorithm 1, where $t^{\mathrm{mep}}$ is the maximal number of epochs.
**Algorithm 1:** TD3-based offloading (TD3O)1: Initialization: $\theta^{Q_{1}}$, $\theta^{Q_{2}}$, $\theta^{\mu }$, $\theta^{Q_{1}^{-}}$, $\theta^{Q_{2}^{-}}$, $\theta^{\mu^{-}}$, $t^{\mathrm{step}}=0$, $t^{\mathrm{epoch}}=0$.2: **While** $t^{\mathrm{epoch}}<t^{\mathrm{mep}}$3:   Let t=0, state $s_{t}$ and reward R=0.4:   **While** t<T5:    Generate action $a_{t}$ using (14).6:    Achieve actual action by executing Algorithm 2.7:    Calculate reward $w_{t}$ using (12) and obtain the state $s_{t+1}$.8:    **If** $t^{\mathrm{step}}\geq \kappa$9:      Replace the previous quadruple with $\left(s_{t},a_{t},w_{t},s_{t+1}\right)$.10:     **Else**11:       Put the quadruple $\left(s_{t},a_{t},w_{t},s_{t+1}\right)$ into the queue.12:     **EndIf**13:     Update state $s_{t}=s_{t+1}$.14:     **If** $t^{\mathrm{step}}\%t^{\mathrm{cti}}=0$ and $t^{\mathrm{step}}>N$15:       Extract *N* quadruples for training.16:       For any sample *n*, $Q_{1}^{-}$ and $Q_{2}^{-}$ networks output ${\tilde{q}}_{n,1}$ and17:        ${\tilde{q}}_{n,2}$, respectively, and obtain the minimum value ${\tilde{q}}_{n}$.18:       Calculate $L(\theta_{1}^{Q})$ and $L(\theta_{2}^{Q})$ using (17) and (18), respectively.19:       Calculate Q gradient using (19) and (20), and clip it.20:       Find $\theta^{Q_{1}}$ and $\theta^{Q_{2}}$ using Adam optimizer.21:       **If** $t^{\mathrm{step}}\%t^{\mathrm{pti}}=0$22:        Calculate *q* through $Q_{1}$.23:        Calculate policy gradient using (16), and clip it.24:        Find $\theta^{\mu }$ using Adam optimizer.25:       **EndIf**26:       Calculate $\theta^{Q_{1}^{-}}$, $\theta^{Q_{2}^{-}}$ and $\theta^{\mu^{-}}$ using (21)–(23), respectively.27:     **EndIf**28:     $R=R+\gamma w_{t}$.29:     $t^{\mathrm{step}}=t^{\mathrm{step}}+1$; t=t+1.30:   **EndWhile**31:     $t^{\mathrm{epoch}}=t^{\mathrm{epoch}}+1$.32: **EndWhile**
**Algorithm 2:** Action transformation (AT)1: **For** each MD m∈M2:   Achieve MD association matrix X using discretization rule.3:   Achieve task caching matrix Y using discretization rule.4:   Achieve task offloading matrix U using discretization rule.5: **EndFor**6: **For** each BS i∈I7:  Returns the set $K_{i}$ of available subchannels and the set $M_{i}$ of8:   offloading MDs.9:  **If** $M_{i}>K_{i}$10:     $M_{i}-K_{i}$ associated MDs are randomly selected, disassociated11:      and execute tasks locally.12:   **EndIf**13:   Achieve subchannel allocation matrix Z using discretization rule.14: **EndFor**15: **For** each MD m∈M16:   **If** $\sum_{i\in I}u_{m,i}=1$17:     **If** ${\bar{f}}_{m}=0$18:      Assign small enough computing capacity to MD *m* to avoid19:       zero division.20:     **Else**21:      Allocate computing resources to MD *m* using (28).22:     **EndIf**23:   **EndIf**24: **EndFor**


### 4.3. AT Algorithm

In order to apply the TD3O algorithm to solve the problem P1, it is necessary to convert the achieved continuous action $a_{t}=\left\{\bar{X}\left(t\right),\bar{Y}\left(t+1\right),\bar{Z}\left(t\right),\bar{U}\left(t\right),\bar{F}\left(t\right)\right\}$ into a discrete one [44]. To this end, we consider the following transformations for $a_{t}$.

#### 4.3.1. The Discretization of Device Association Array

In $\bar{X}=\left\{{\bar{x}}_{m}\left|\forall m\in M\right.\right\}$, ${\bar{x}}_{m}$ is the non-integer association index of MD *m*, which is the continuous action achieved by the TD3 algorithm. Then, it is converted into an integer form, i.e.,

(24) $$\left\{\begin{array}{c} x_{m,\mathrm{ceil}\left(I{\bar{x}}_{m}\right)}=1,\;\mathrm{if}\;I{\bar{x}}_{m}\neq 0,\\ x_{m,1}=1,\;\mathrm{otherwise}, \end{array}\right.$$

where ceil(b) is an upward rounding function with respect to *b*. Such a transformation can ensure that each MD can be associated with one BS.

#### 4.3.2. The Discretization of Task-Caching Array

In $\bar{Y}$, ${\bar{y}}_{m}$ represents the non-integer caching index of MD *m*, which is the continuous action achieved by the TD3 algorithm. Since each MD can store its task at all BSs, there exist $2^{I}$ storage options for it. Consequently, in order to convert ${\bar{y}}_{m}$ into a discrete form, we first need to perform

(25) $$\left\{\begin{array}{c} {\hat{y}}_{m}=\mathrm{floor}\left(2^{I}{\bar{y}}_{m}\right),\;\mathrm{if}\;\;2^{I}{\bar{y}}_{m}\neq 0,\\ {\hat{y}}_{m}=0,\;\mathrm{otherwise}, \end{array}\right.$$

where floor(b) is a downward rounding function with respect to *b*. Then, in order to achieve the binary caching index, the decimal ${\hat{y}}_{m}$ needs to be converted into a binary number of *I* 0–1 digits, which is given by $\mathrm{bin}\left({\bar{y}}_{m}\right)$. In it, bin(b) is a function used for calculating the binary number of decimal *b*. Then, $y_{m,i}=\mathrm{bin}{\left({\bar{y}}_{m}\right)}_{i}$, where $\mathrm{bin}{\left({\bar{y}}_{m}\right)}_{i}$ represents the *i*-th digit of the binary number $\mathrm{bin}\left({\bar{y}}_{m}\right)$.

#### 4.3.3. The Discretization of Task Offloading Array

In $\bar{U}$, ${\bar{u}}_{m}$ is the non-integer offloading index of MD *m*, which is the continuous action achieved by the TD3 algorithm. Considering that each MD can offload its task to at most one BS, ${\bar{u}}_{m}$ is converted into an integer form, i.e.,

(26) $$\left\{\begin{array}{c} u_{m,\mathrm{ceil}\left(I{\bar{u}}_{m}\right)}=1,\;\mathrm{if}\;I{\bar{u}}_{m}\neq 0,\\ u_{m,i}=0,\forall i\in I,\;\mathrm{otherwise}. \end{array}\right.$$

#### 4.3.4. The Discretization of Subchannel Allocation Array

In $\bar{Z}$, ${\bar{z}}_{i}$ is the non-integer index of the subchannels allocated by BS *i* to its associated MDs who need to offload tasks, which is the continuous action achieved by the TD3 algorithm. To achieve the integer form of ${\bar{z}}_{i}$, we first need to perform

(27) $$\left\{\begin{array}{c} {\hat{z}}_{i}=\mathrm{ceil}\left(C\left(M_{i},K_{i}\right){\bar{z}}_{i}\right),\;\mathrm{if}\;C\left(M_{i},K_{i}\right){\bar{z}}_{i}\neq 0,\\ {\hat{z}}_{i}=1,\;\mathrm{otherwise}, \end{array}\right.$$

where $M_{i}$ is the number of MDs that are associated with BS *i* and need to offload tasks; $K_{i}$ is the number of available subchannels at BS *i*; $C\left(M_{i},K_{i}\right)=\mathrm{fac}\left(K_{i}\right)/\mathrm{fac}\left(M_{i}\right)\mathrm{fac}\left(K_{i}-M_{i}\right)$ is a function with respect to $M_{i}$ and $K_{i}$ and is used for calculating the number of feasible subchannel allocation policies between $M_{i}$ MDs and $K_{i}$ subchannels at BS *i*; fac(b) is a factorial function with respect to *b*; $M_{i}\leq K_{i}$ shall be satisfied.

Then, we assume that $Z_{i}=\left\{1,2,\cdots ,C\left(M_{i},K_{i}\right)\right\}$ is the set of $C\left(M_{i},K_{i}\right)$ feasible subchannel allocation policies between $M_{i}$ MDs and $K_{i}$ subchannels at BS *i*. After that, the subchannel allocation policy ${\hat{z}}_{i}$ in the set $Z_{i}$ is selected according to the Equation (27). It is noteworthy that $C\left(M_{i},K_{i}\right)$ feasible subchannel allocation policies are generated in advance. That is to say, in the policy ${\hat{z}}_{i}$, we can easily know the utilized indices of $K_{i}$ subchannels for $M_{i}$ MDs. According to these rules, we can easily find the subchannel allocation index Z.

#### 4.3.5. The Transformation of Computing Resource Allocation Array

In $\bar{F}=\left\{{\bar{f}}_{m}\left|\forall m\in M\right.\right\}$, ${\bar{f}}_{m}$ represents the computing resource score of MD *m* at the target BS that is executing its task. If $\sum_{i\in I}u_{m,i}=1$ is satisfied between MD *m* and BS *i*, according to the proportional allocation of computing resources, the computing resources allocated to MD *m* by BS *i* can be given by

(28) $$f_{m,i}=u_{m,i}f_{i}^{\mathrm{BS}}{\bar{f}}_{m}/\sum_{j\in M}u_{j,i}{\bar{f}}_{j}.$$

Based on the above-mentioned operations, the output action $a_{t}=\left\{\bar{X},\bar{Y},\bar{Z},\bar{U},\bar{F}\right\}$ of the TD3O algorithm can be effectively converted into an actual decision, which is summarized as Algorithm 2.

### 4.4. HA

To solve the problem P1 in a non-iterative manner, we design an effective heuristic algorithm, which is summarized in Algorithm 3. In such an algorithm, to reduce the uplink transmission time and energy consumption, some MDs are associated with the nearest BSs, and the BSs randomly cache the tasks of their associated MDs until the cache space cannot cache more tasks. Then, the uncached MDs randomly select a BS as the offloading target, and to guarantee time constraints, the BS will evenly distribute the computing resources according to the computation amount of the task. Finally, a part of the MDs are disassociated from BSs without sufficient subchannels and execute tasks by themselves.
**Algorithm 3:** Heuristic algorithm (HA)1: Initialization: energy consumption ${\bar{\varepsilon }}^{\mathrm{tot}}=0$.2: Each MD selects (is associated with) the nearest BS.3: **For** each BS i∈I4:    **If** $M_{i}>K_{i}$5:     $M_{i}-K_{i}$ associated MDs are randomly selected, disassociated6:      and execute tasks locally.7:    **EndIf**8:    Randomly select the tasks of MDs associated with BS *i* for caching9:     until the caching space is full.10: **EndFor**11: **For** t∈T12:    Randomly select a target BS for each MD without cached task.13:    Randomly allocate subchannels to MDs associated with each BS.14:    **If** subchannels are insufficient15:     Extra MDs are randomly selected to execute tasks locally.16:    **EndIf**17:    Proportionally allocate computing resources to MDs associated with18:     each BS according to the CPU cycles required by tasks.19:    Calculate the total local energy consumption $\bar{\varepsilon }$.20:    ${\bar{\varepsilon }}^{\mathrm{tot}}={\bar{\varepsilon }}^{\mathrm{tot}}+\bar{\varepsilon }$.21: **EndFor**

## 5. Algorithm Analysis

### 5.1. Computation Complexity Analysis

In this section, the computation complexity of proposed algorithms are analysed as follows.

**Proposition** **1.** *The computation complexity of Algorithm 2 is $O\left(MIK\right)$ in the worst case.*

**Proof.**  In Algorithm 2, the computation complexity of steps 1–5 is $O\left(M\right)$, the computation complexity of steps 6–14 is $O\left(MIK\right)$ in the worst case, and the computation complexity of steps 15–24 is $O\left(MI\right)$. In general, the computation complexity of Algorithm 2 is $O\left(MIK\right)$ in the worst case. □

**Proposition** **2.** *The computation complexity of Algorithm 1 is $O(\max(\sum_{l=0}^{L^{Q}}\psi_{l}^{Q}\psi_{l+1}^{Q},\sum_{l=0}^{L^{\mu }}$* *$\psi_{l}^{\mu }\psi_{l+1}^{\mu }))$ at each timeslot, where $L^{\mu }$ is the number of layers of the policy network, $L^{Q}$ is the number of layers of the critic network, $\psi_{l}^{\mu }$ is the number of neurons at the l-th layer of the policy network and $\psi_{l}^{Q}$ is the number of neurons at l-th layer of the critic network.*

**Proof.**  In Algorithm 1, the computation complexity is mainly related to the action transformation, the calculation of the reward and task processing time and the structure of the neural network. As previously mentioned, the computation complexity of the action transformation should be $O\left(MIK\right)$ in the worst case. As seen from Formulas (8) and (12), the computation complexity of the calculation of the reward and task processing time is $O\left(MIK\right)$.

In Algorithm 1, there exist four critic networks and two policy networks. We assume that the structure of the policy network and its target network is the same, and the structure of the two critic networks and its target network is the same. Then, we can easily deduce that the computation complexity of establishing policy networks is $O\left(\sum_{l=0}^{L^{\mu }}\psi_{l}^{\mu }\psi_{l+1}^{\mu }\right)$ and the computation complexity of establishing critic networks is $O\left(\sum_{l=0}^{L^{Q}}\psi_{l}^{Q}\psi_{l+1}^{Q}\right)$. Therefore, the computation complexity of establishing neural networks is $O(\max(\sum_{l=0}^{L^{Q}}\psi_{l}^{Q}\psi_{l+1}^{Q}$, $\sum_{l=0}^{L^{\mu }}\psi_{l}^{\mu }$$\psi_{l+1}^{\mu }))$.

Since the computation complexity of establishing neural networks is much higher than that of the other operations in Algorithm 2. In general, the computation complexity of Algorithm 2 is $O\left(\max\left(\sum_{l=0}^{L^{Q}}\psi_{l}^{Q}\psi_{l+1}^{Q},\sum_{l=0}^{L^{\mu }}\psi_{l}^{\mu }\psi_{l+1}^{\mu }\right)\right)$ at each timeslot. □

**Proposition** **3.** *The computation complexity of Algorithm 3 is $O\left(MI\right)$ at each timeslot.*

**Proof.**  In Algorithm 3, the computation complexity of step 2 is $O\left(MI\right)$, the computation complexity of steps 3–10 is $O\left(I\right)$, the computation complexity of steps 12–16 is $O\left(M\right)$, the computation complexity of steps 17–19 is $O\left(MI\right)$. In general, the computation complexity of Algorithm 3 is $O\left(MI\right)$ at each timeslot. □

### 5.2. Convergence Analysis

Since Algorithm 2 is a part of Algorithms 1 and 3 and is non-iterative, we just need to concentrate on the convergence of Algorithm 1. In detail, this is established as follows.

**Theorem** **1.** *Algorithm 1 can be guaranteed to converge after finite iterations.*

**Proof.**  In Algorithm 1, the neural networks are updated by the gradient descent method used in the Adam optimizer. This utilizes the gradient information of the functions $J(\theta^{\mu })$, $L_{1}\left(\theta^{Q_{1}}\right)$ and $L_{2}\left(\theta^{Q_{2}}\right)$ to guide the updating directions of the parameters $\theta^{\mu }$, $\theta^{Q_{1}}$ and $\theta^{Q_{2}}$, so that the objective functions can reach the optimal or suboptimal values. When these values tend to be stable, the parameters $\theta^{\mu }$, $\theta^{Q_{1}}$ and $\theta^{Q_{2}}$ also tend to be stable. At this time, Algorithm 1 is deemed convergent. □

## 6. Performance Evaluation

In order to verify the performance of the designed algorithms, we introduce the following algorithms for comparison.

DDPG-based offloading (DDPGO): DDPG is a classical DRL algorithm [45]. Compared with the TD3 algorithm, the DDPG algorithm reduces the critic network and the critic target network. In addition, both the critic network and policy network are updated at each timeslot in the DDPG algorithm. In this paper, the DDPG algorithm used to solve the problem P1 is named the DDPG-based offloading (DDPGO) algorithm. The difference between the two algorithms is that the state input and action output are the same as the mode used by TD3O, and it also uses the AT algorithm to convert continuous actions.

Completely offloading (CO): In the CO algorithm, the task of each MD is offloaded to the nearest BS for computing. Such BS proportionally allocates the computing capacity to its associated MDs according to the CPU cycles required by the tasks of these MDs.

Completely local executing (CLE): In the CLE algorithm, the tasks of all MDs can be executed by themselves.

In this paper, we consider that each BS is deployed in a non-overlapping area with a radius of 400 m and a power spectral density of −174 dBm/Hz. In addition, I=3, $f_{m}^{\mathrm{loc}}=1\;\mathrm{GHz}$, $f_{i}=8\;\mathrm{GHz}$, W=40MHz, K=4, $d_{m}=2$∼5MB, $c_{m}=50\;\mathrm{cycles}/\mathrm{bit}$, $\xi =10^{-27}$, $\tau_{m}=10\;s$, ρ=2, $r^{\mathrm{bh}}=1\;\mathrm{Gbps}$, $p_{m}=23\;\mathrm{dBm}$, κ=80,000, N=128, γ=0.94 and λ=0.04. In the DRL algorithm, we consider that both the policy network and the critic network are composed of three-layer fully connected neural networks, where the numbers of neurons in three-layer neural networks in the policy network are 300, 200 and 128, respectively, and the corresponding target network has the same structure with this policy network; the number of neurons in three-layer neural networks in the critic network are 300, 128 and 32, respectively, and the corresponding target network has the same structure as this critic network. Significantly, the first-layer fully connected neural network of the policy network and the critic network utilizes the rectified linear unit 6 (RELU6), which suppresses the maximum value as the activation function, while other layers use RELU as the activation function.

**Figure 5:**
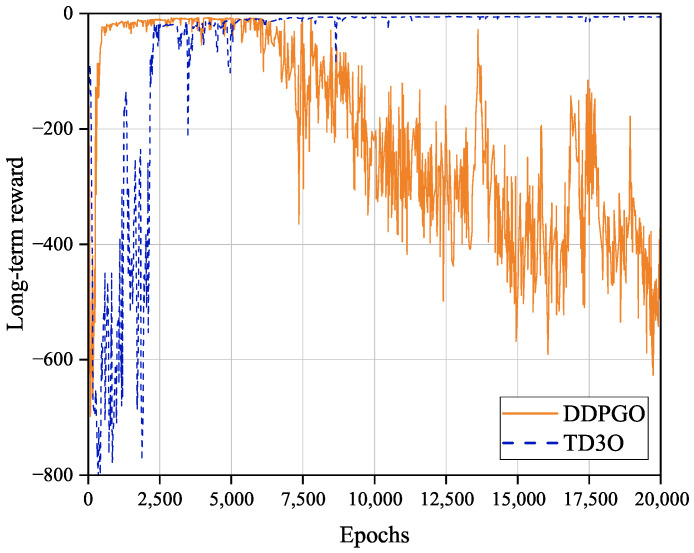
The convergence of DDPGO and TD3O algorithms.

Figure 5 shows the convergence of the TD3O and DDPGO algorithms. As shown in Figure 5, DDPGO may have a higher convergence rate than TD3O, but the former may have worse convergence stability than the latter. The reason for this may be that the critic network and the policy network are updated synchronously in DDPGO. In DDPGO, the network parameters are updated in each training phase, which speeds up the convergence. Synchronously, the policy network parameters are updated in the training, which results in the instability of the long-term reward value and training bias. As we know, TD3O is composed of two sets of critic networks. Consequently, it could be trained in a relatively stable Q value so that the algorithm can converge stably. In the simulation, it is also easy to find that TD3O could achieve a more stable and better solution to the problem P1 than DDPGO in general.

**Figure 6:**
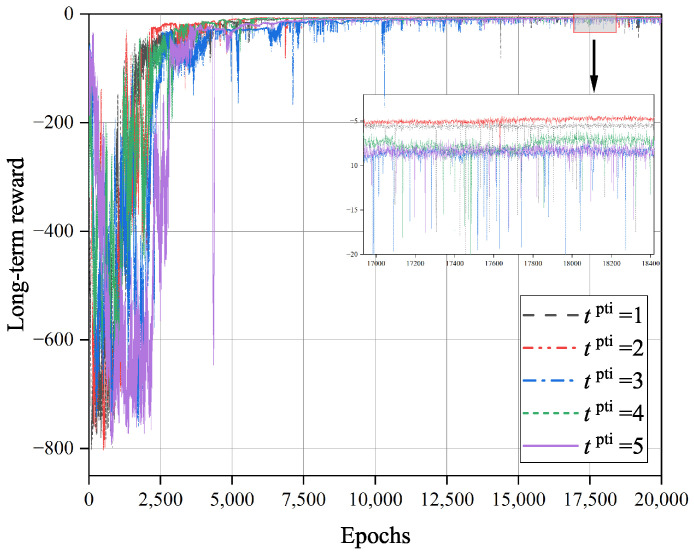
The impact of training interval $t^{\mathrm{pti}}$ on the convergence of TD3O algorithm.

Figure 6 shows the impact of the training interval $t^{\mathrm{pti}}$ on the convergence of the TD3O algorithm. As we know, under the same number of iterations, a larger $t^{\mathrm{pti}}$ can effectively reduce the overall training time of the network. However, it will reduce the total learning times of the policy network and its target network. As illustrated in Figure 6, the convergence rate of TD3O may decrease with $t^{\mathrm{pti}}$ in general.

**Figure 7:**
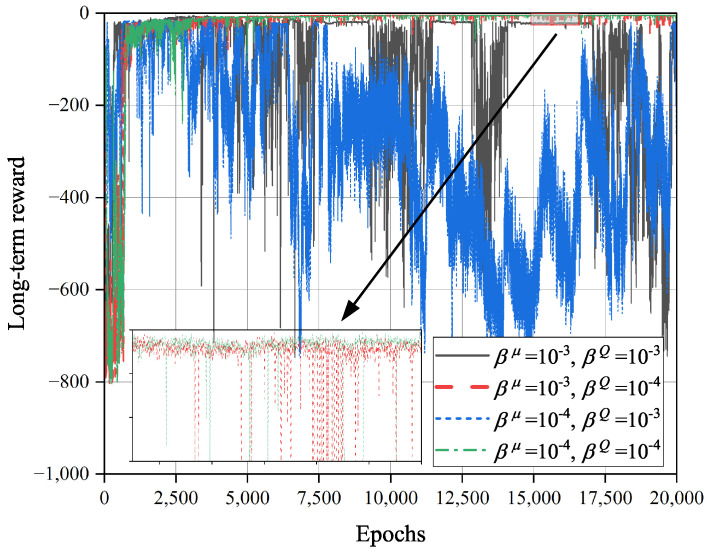
The impacts of learning rates $\beta^{Q}$ and $\beta^{\mu }$ on the convergence of TD3O algorithm.

Figure 7 shows the impacts of learning rates $\beta^{Q}$ and $\beta^{\mu }$ on the convergence of the TD3O algorithm. As we know, when the learning rate $\beta^{Q}$ of the critic network increases, the parameters of such network will be updated at a larger scale, which speeds up the convergence of TD3O. However, it may lead to the failure of stable evaluation of environmental information, which weakens the convergence stability of TD3O. As illustrated in Figure 7, when $\beta^{Q}=0.001$, the convergence rate of TD3O is relatively high, but the achieved long-term reward dramatically fluctuates at this moment. On the other hand, the learning rate $\beta^{\mu }$ of the policy network can affect the optimization capability of TD3O. Specifically, a lower $\beta^{\mu }$ means a smaller amplitude of updating the policy network, which is better for finding better solutions. As seen from Figure 7, TD3O can achieve a better long-term reward when $\beta^{Q}=0.0001$ and $\beta^{\mu }=0.0001$.

**Figure 8:**
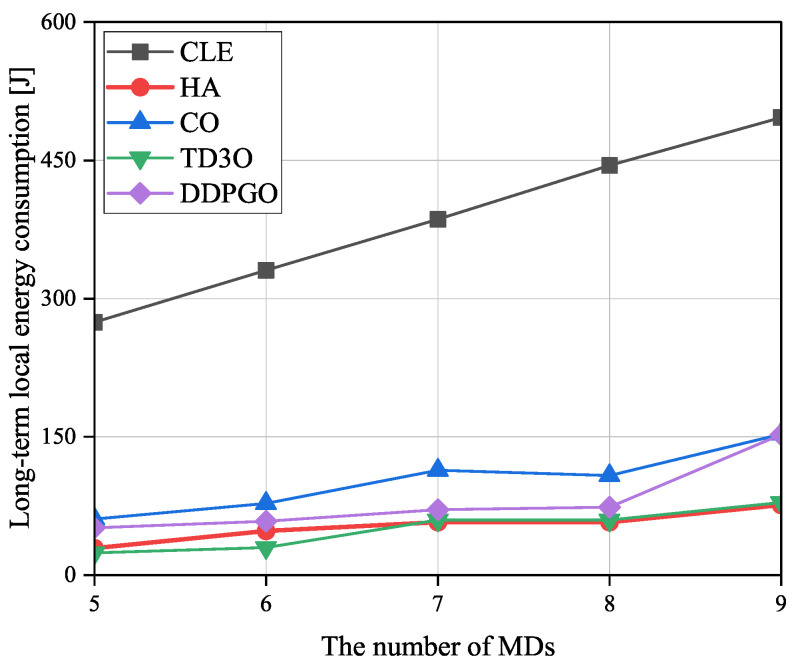
The impacts of the number of MDs on the long-term local energy consumption $\varepsilon^{\mathrm{MD}}$.

Figure 8 shows the impact of the number of MDs on the long-term local energy consumption $\varepsilon^{\mathrm{MD}}$, where $\varepsilon^{\mathrm{MD}}$ is the sum of the total local energy consumption in *T* timeslots. In general, the $\varepsilon^{\mathrm{MD}}$ increases with the number of MDs since a greater energy consumption is used when tackling more tasks of more MDs. Since CLE executes tasks in maximal computation capacity, it could achieve the highest $\varepsilon^{\mathrm{MD}}$ among all algorithms. In CO, MDs are associated with the nearest BSs, which may result in a relatively imbalanced load distribution. Then, some overloaded BSs cannot provide good services for their associated MDs because of limited resources, which may result in high $\varepsilon^{\mathrm{MD}}$. Consequently, CO could achieve higher $\varepsilon^{\mathrm{MD}}$ than other algorithms excluding CLE. As illustrated in Figure 8, TD3O could achieve lower $\varepsilon^{\mathrm{MD}}$ than DDPGO since the former can effectively mitigate the overestimation existing in the latter. Although HA lets MDs be associated with the nearest BSs, some MDs associated with overloaded BSs will disassociate and execute tasks locally. Such an operation may result in relatively low $\varepsilon^{\mathrm{MD}}$.

**Figure 9:**
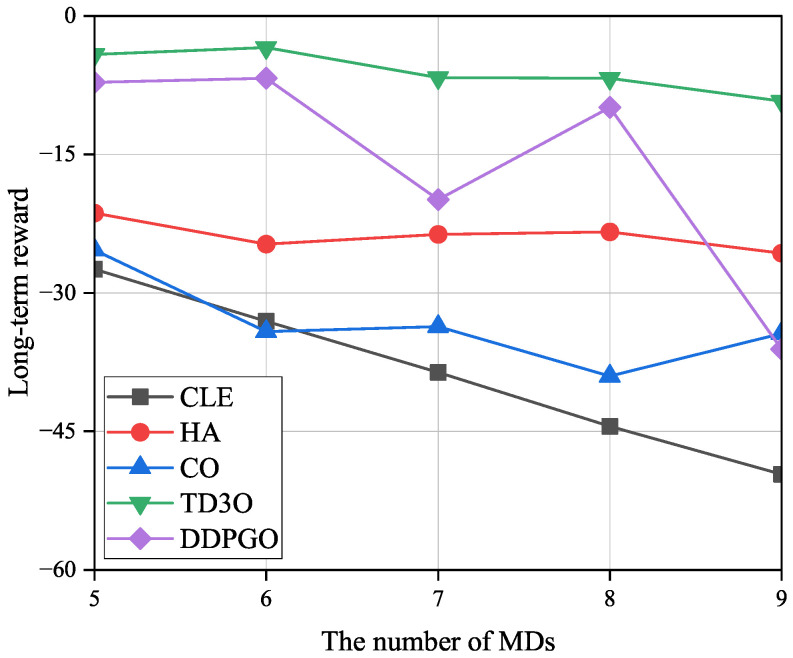
The impacts of the number of MDs on the long-term reward *R*.

Figure 9 shows the impacts of the number of MDs on the long-term reward (*R*). As illustrated in Figure 9, *R* may decrease with the number of MDs since more MDs result in a higher energy consumption. Since both TD3O and DDPGO try to maximize the reward but in other algorithms this is not the case, the former could achieve higher *R* than other algorithms in general. Since TD3O could achieve lower $\varepsilon^{\mathrm{MD}}$ than DDPGO, the former could achieve a higher *R* than the latter. In view of the unstable convergence of DDPGO, its reward may dramatically fluctuate. Since CLE could achieve the highest $\varepsilon^{\mathrm{MD}}$ among all algorithms, it could achieve the lowest *R* in general. In addition, CO could achieve a lower *R* than HA since the former consumes more energy than the latter.

**Figure 10:**
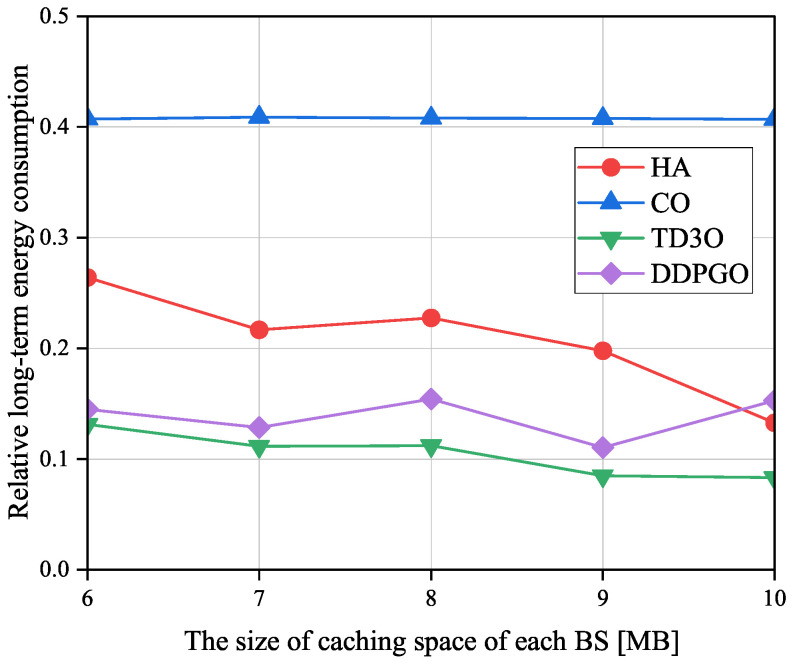
The impacts of the size of caching space of each BS on the relative long-term energy consumption $\eta^{\varepsilon }$.

Figure 10 shows the impacts of the size of caching space of each BS on the relative long-term energy consumption $\eta^{\varepsilon }$ denoted as the ratio of $\varepsilon^{\mathrm{MD}}$ achieved by an offloading algorithm to the one attained by CLE. Evidently, a smaller $\eta^{\varepsilon }$ means a higher energy gain caused by offloading tasks. As illustrated in Figure 10, in addition to DDPGO and CO, $\eta^{\varepsilon }$ in other algorithms decreases with the size of the caching space of each BS in general. The reason for this may be that a larger caching space can hold more tasks to reduce the transmission energy consumption. However, as revealed in Figure 5 and Figure 9, the unstable convergence of DDPGO may result in a dramatically fluctuating performance. Therefore, $\eta^{\varepsilon }$ in DDPGO may evidently be fluctuating. In addition, $\eta^{\varepsilon }$ in CO may not change with the size of caching space of each BS since it does not utilize caching space. By minimizing the $\varepsilon^{\mathrm{MD}}$, TD3O and DDPGO could achieve a lower $\eta^{\varepsilon }$ than other algorithms in general. In addition, TD3O could achieve a lower $\eta^{\varepsilon }$ than DDPGO since the former mitigates the overestimation existing in DDPGO. As seen from Figure 10, CO could achieve the highest $\eta^{\varepsilon }$ among all algorithms since it has no sufficient resources to provide for MDs associated with overloaded BSs.

**Figure 11:**
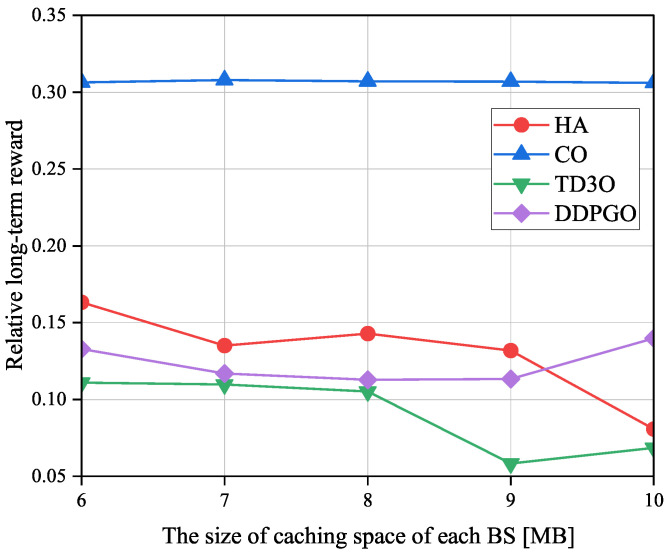
The impacts of the size of caching space of each BS on the relative long-term reward $\eta^{R}$.

Figure 11 shows the impacts of the size of the caching space of each BS on the relative long-term reward $\eta^{R}$ denoted as the ratio of the long-term reward achieved by an offloading algorithm to the one attained by CLE. Evidently, a smaller $\eta^{R}$ means a higher reward gain caused by offloading tasks. As illustrated in Figure 11, $\eta^{R}$ in TD3O and HA decreases with the size of the caching space of each BS in general. The reason for this may be that a larger caching space can hold more tasks to reduce the transmission energy consumption, and then bring a higher reward. However, due to the unstable convergence of DDPGO, $\eta^{R}$ in DDPGO may be fluctuating. Moreover, $\eta^{R}$ in CO may not change with the size of caching space of each BS since it does not utilize caching space. By minimizing the $\varepsilon^{\mathrm{MD}}$ and thus increasing the reward, TD3O and DDPGO could achieve a lower $\eta^{R}$ than other algorithms in general. In addition, TD3O could achieve a lower $\eta^{R}$ than DDPGO since the former mitigates the overestimation existing in DDPGO. As can be seen from Figure 11, CO could achieve the highest $\eta^{R}$ because of the high energy consumed by MDs associated with overloaded BSs.

As seen from the above-mentioned simulation figures, although HA is a non-iterative algorithm, it could sometimes achieve better performance than DDPGO. In addition, it may always achieve fairly better performance than CO and CLE.

## 7. Conclusions

In this paper, the problem of minimizing the local energy consumption is concentrated in the cache-assisted vehicular NOMA–MEC networks under time and resource constraints, which refers to the joint optimization of the computing resource allocation, subchannel selection, device association, offloading and caching decisions. To solve the formulated problem, we developed an effective TD3O algorithm that was integrated with the AT algorithm and designed HA simultaneously. As for the designed algorithms, we have given some analyses of the convergence and computation complexity. Simulation results show that TD3O could achieve lower local energy consumption than several benchmark algorithms, and HA could achieve lower local energy consumption than the CO and CLE algorithms. Future work can include power allocation and secure communications, such as optimizing the transmission power of MDs for task offloading, and how to encrypt part of the task data in the network at a low cost to achieve secure communication, etc.

## Data Availability

Data are contained within the article.
